# Novel Dominant KCNQ2 Exon 7 Partial In-Frame Duplication in a Complex Epileptic and Neurodevelopmental Delay Syndrome

**DOI:** 10.3390/ijms21124447

**Published:** 2020-06-23

**Authors:** Pedro A. Lazo, Juan L. García, Paulino Gómez-Puertas, Íñigo Marcos-Alcalde, Cesar Arjona, Alvaro Villarroel, Rogelio González-Sarmiento, Carmen Fons

**Affiliations:** 1 Molecular Mechanisms of Cancer Program, Instituto de Biología Molecular y Celular del Cáncer, Consejo Superior de Investigaciones Científicas (CSIC), Universidad de Salamanca, 30007 Salamanca, Spain; jlgarcia@usal.es (J.L.G.); gonzalez@usal.es (R.G.-S.); 2 Instituto de Investigación Biomédica de Salamanca (IBSAL), Hospital Universitario de Salamanca, 30007 Salamanca, Spain; 3 Centro de Biología Molecular Severo Ochoa, CSIC-Universidad Autónoma de Madrid, Cantoblanco, E-28049 Madrid, Spain; pagomez@cbm.csic.es (P.G.-P.); imarcos@cbm.csic.es (Í.M.-A.); 4 Biosciences Research Institute, School of Experimental Sciences, Universidad Francisco de Vitoria, 28223 Madrid, Spain; 5 Institut de Recerca Sant Joan de Déu, Esplugues de Llobregat, 08950 Barcelona, Spain; carjona@personalgenetics.cat (C.A.); cfons@sjdhospitalbarcelona.org (C.F.); 6 Instituto Pediátrico de Enfermedades Raras (IPER), Hospital Sant Joan de Déu, 08950 Barcelona, Spain; 7 Instituto de Biofísica, Consejo Superior de Investigaciones Científicas (CSIC), Universidad del País Vasco, 48940 Bilbao, Spain; alvaro.villarroel@csic.es; 8 Unidad de Genética Molecular, Departamento de Medicina, Universidad de Salamanca, 37008 Salamanca, Spain; 9 Neurology Department, Hospital Sant Joan de Déu, Sant Joan de Déu Research Institute and CIBERER, Instituto de Salud Carlos III, 08950 Barcelona, Spain

**Keywords:** epilepsy, dystonia, cerebral palsy, neuromotor delay, KCNQ2

## Abstract

Complex neurodevelopmental syndromes frequently have an unknown etiology, in which genetic factors play a pathogenic role. This study utilizes whole-exome sequencing (WES) to examine four members of a family with a son presenting, since birth, with epileptic-like crises, combined with cerebral palsy, severe neuromotor and developmental delay, dystonic tetraparexia, axonal motor affectation, and hyper-excitability of unknown origin. The WES study detected within the patient a de novo heterozygous in-frame duplication of thirty-six nucleotides within exon 7 of the human KCNQ2 gene. This insertion duplicates the first twelve amino acids of the calmodulin binding site I. Molecular dynamics simulations of this KCNQ2 peptide duplication, modelled on the 3D structure of the KCNQ2 protein, suggest that the duplication may lead to the dysregulation of calcium inhibition of this protein function.

## 1. Introduction

Newborns with severe non-hereditary neuromotor and neurodevelopmental delays, cerebral palsy, and epileptic crises often have an unknown etiology, which are likely to have a heterogeneous origin, in which genetic alterations can play a relevant pathogenic role.

Voltage-gated sodium channels generate neuronal action potentials [1]. Voltage-gated potassium channels re-establish these potentials back to normal [2], and the functional coordination of these channels regulate the neuronal excitability, controlling neuronal networks [3]. These two types of ion channels have specific subcellular locations on neuron membranes [4]. The pathogenesis of severe early neuromotor developmental delays and epilepsy is associated with mutations or variants in several genes. Among them, KCNQ2 forms dimers with KCNQ3 in order to assemble the potassium M channel [5], which limits repetitive firing [6,7], controlling the excitability of neurons [8] and their responsiveness to synaptic inputs [9]. Human KCNQ2 pathogenic variants have been associated to benign familial neonatal epilepsy/infantile epileptic encephalopathy type 7 (BFNS, OMIM: 121200) and to early onset epileptic encephalopathy (EOEE; OMIM: 613720), but as the number of cases increases, the associated clinical phenotypes are becoming more heterogeneous. Known KCNQ2 pathogenic variants are distributed throughout the length of the protein, with two-thirds located within trans-membrane domains. However, there is no correlation between the location of the variant amino acid on the protein sequence, the type of pathogenic variant, and the severity of the neurological phenotype. This variability suggests that KCNQ2 alterations are necessary for epileptic syndromes [10]. However, the severity and variability of the associated full neurological phenotype is very likely determined by the contribution of additional genetic alterations or variants not yet identified [10,11,12]. The clinical heterogeneity of these neurological syndromes reflects a very complex situation in which an unknown combination of alterations in gene dosage and pathogenic variants may contribute to the pathogenesis of complex neurodevelopmental phenotypes, including epileptic disorders [11,13,14,15], cerebral palsy [16,17], and autism [18,19], among others.

## 2. Results

### 2.1. Epileptic, Cerebral Palsy, and Neuromotor Developmental Delay Phenotype of Patient

**Figure 1:**
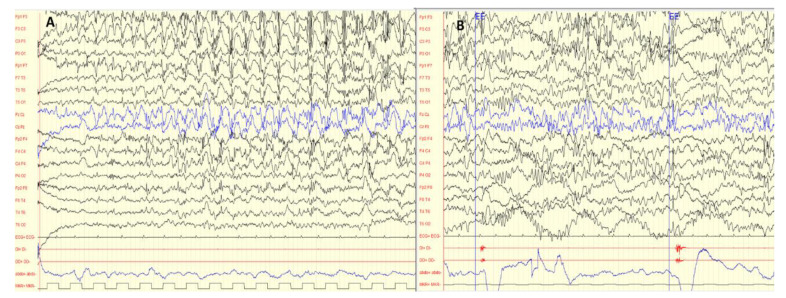
Electroencephalogram (EEG) of the patient at six years of age. (**A**) Interictal EEG. Bilateral polyspike-and-wave discharges with frontal predominance. (**B**) Multifocal epileptiform abnormalities.

The case is a 6-year-old boy presenting with epileptic encephalopathy of neonatal onset, cerebral palsy, and a severe neuromotor delay of unknown origin. Pregnancy with gestational diabetes was controlled with diet. Delivery was uneventful. After 48 h of life, the boy presented with episodes of cyanosis, generalized hypertonia, and tonic asymmetric postures followed by apnea. Video-EEG at 5 days of life showed bilateral and asynchronous spike-and-wave. Seizures were refractory to phenobarbital, but were controlled with phenytoin. At 3 months of age, the boy presented with startle episodes without EEG correlation related to the wake and sleep transition, together with dystonic postures. One month later, epileptic spasms without hypsarrhythmia were observed. There was no response to levetiracetam and valproic acid, but spasms stopped after lacosamide (LCS) treatment was initiated (11 months of age). Metabolic tests (including cerebro-spinal fluid(CSF) studies), karyotype, and brain MRI were normal. The progressive increase of Thyroid stimulating hormone (TSH) was treated with oral L-T4. At 20 months, the boy presented with a recurrence of spasms that were controlled with LCS dose adjustment. After several years of good seizure control, LCS was discontinued. The boy suffered from the decompensation of seizures with a febrile viral infection, and at 5 years of age he presented with startle episodes associated to auditory stimulus without EEG correlation, whereas during sleep he presented with epileptic spasms with EEG correlation. Treatment with vigabatrin was initiated with a clinical and EEG improvement. Video-EEG showed abnormal background activity (Figure 1A) and paroxysmal multifocal discharges that showed activation and generalization during sleep (Figure 1B). The final video-EEG monitoring showed bilateral polyspike discharges and clinical spasms in the polygraph recording, which did not show any clear EEG correlation.

Many KCNQ2 encephalopathy patients present a very good response to sodium channel blockers, as our patient did with phenytoin and LCS during the neonatal period, and later when treatment with LCS was reintroduced.

During a physical exam, the five-year old child presented with a severe axial hypotonia without head control, hypertonic movements of four limbs with dystonic movements of the upper limbs, no hand use, and was not able to sit or crawl. The child had a dystonic tetraparesia and peripheral neuro-axonal motor affectation. The child had no development of expressive language.

### 2.2. Partial de Novo KCNQ2 Exon 7 Duplication

This complex and severe neurodevelopmental syndrome could be due to the phenotypic convergence of several genetic alterations whose biological functions contribute to different aspects of this complex clinical phenotype. The initial approach used to identify them was a whole-exome sequencing (WES) study.

**Figure 2:**
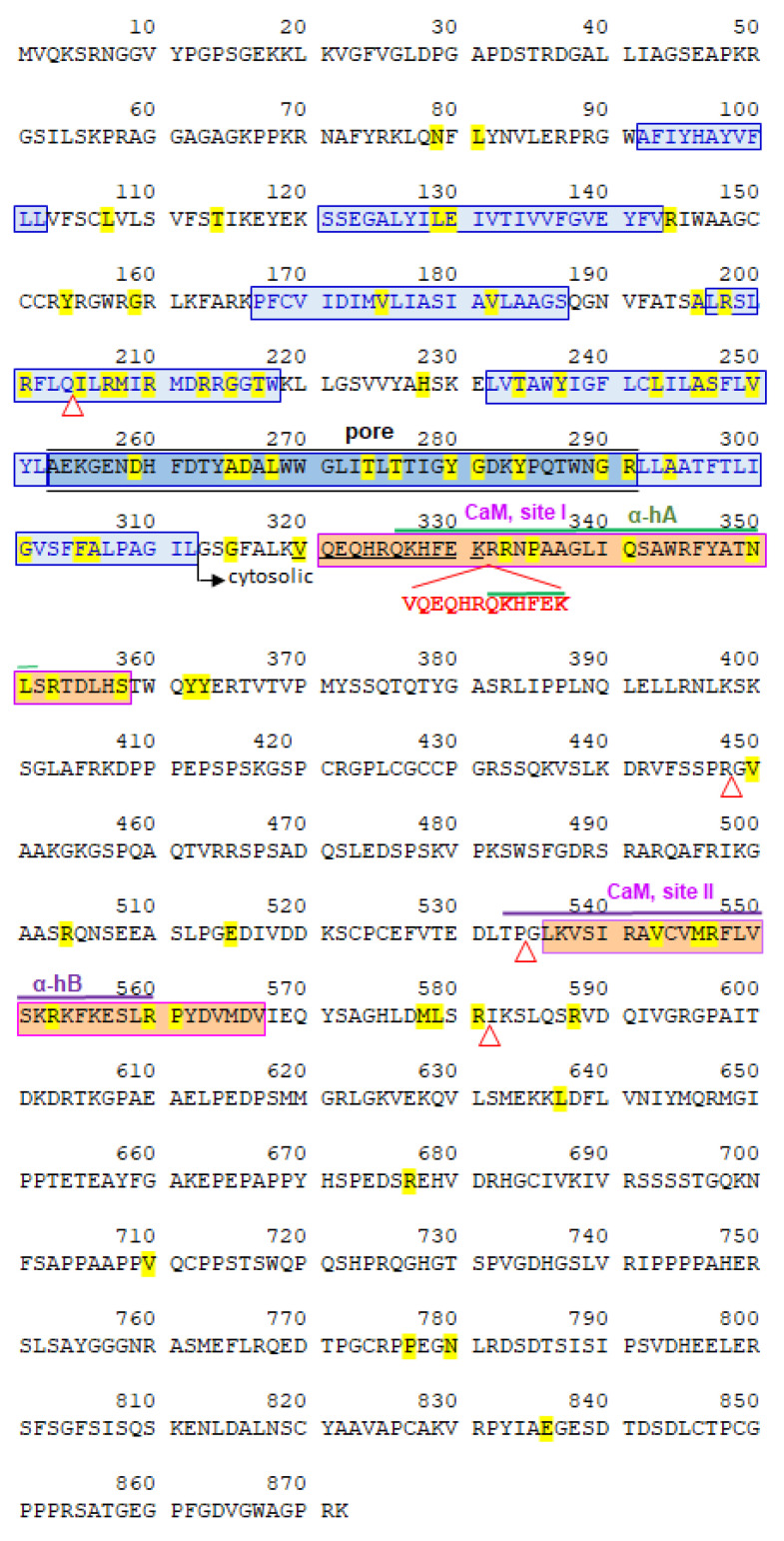
Partial duplication of exon 7 KCNQ2 in the calmodulin interaction site. The twelve duplicated amino acids are underlined, and the inserted peptide is indicated in red. In the sequence, the amino acids in yellow indicate the 73 residues affected by the pathogenic variants reported in 159 different patients obtained from the ClinVar database and publications referenced in the text. The human KCNQ2 protein reference is UniProtKB (O43526-1). The triangles indicate short deletions or insertions that caused a change in reading frame and a truncated protein. Information in blue boxes indicates the transmembrane domain (TM). The pore region is indicated with the black line. Information in purple indicates the two regions that interacted with calmodulin (CaM) that have two alpha helixes (α-hA in green and α-hB in dark purple).

WES detected in heterozygosis a de novo alteration, not present in the parents, in the KCNQ2 gene that was confirmed by Sanger sequencing. This genetic alteration presented as an in-frame sequence duplication (DUP) of thirty-six nucleotides within exon 7 (GRCh37/hg19: chr20:g.62070007_62070008dupCTTCTCAAAGTGCTTCTGCCTGTGCTGCTCCTGAAC; c.958_993dupGTTCAGGAGCAGCACAGGCAGAAGCACTTTGAGAAG), (GRCh38/hg38: chr20:g.63438654_63438655dupCTTCTCAAAGTGCTTCTGCCTGTGCTGCTCCTGAAC), which resulted in the addition of twelve extra amino acids in the protein (NC_000020.11:g.63438659_63438694dup; p.Val320_Lys331 (ValGlnGluGlnHisArgGlnLysHisPheGluLys). This peptide duplication generated a tandem repeat of the first twelve residues (320–331) of the calmodulin binding site 1 (321–358) [20] and was located at the start of the cytosolic regions of KCNQ2, immediately after the end of the last transmembrane domain of the KCNQ2 protein (Figure 2). This peptide duplication was considered pathogenic by Polyphen-2 [21] and VarElect [22] program analysis; it was also considered pathogenic according to the ACMG criteria [23].

The location of the thirty-six nucleotide duplication was confirmed by Sanger sequencing (Supplementary Figure S1). Thus, this case represents the first association of this very rare type of variant to a neurological phenotype. All the genes identified by WES in the patient are listed in Table S1. The WES analysis also detected a RARS2 mutation in the heterozygosis that codes for the mutant p.S443P, which was inherited from the father (Supplementary Figure S2). RARS2 codes for an arginyl tRNA synthetase, and RARS2 mutations have been associated to pontocerebellar hypoplasia [24].

### 2.3. Dynamic Molecular Modelling of KCNQ2 Partial Duplication

**Figure 3:**
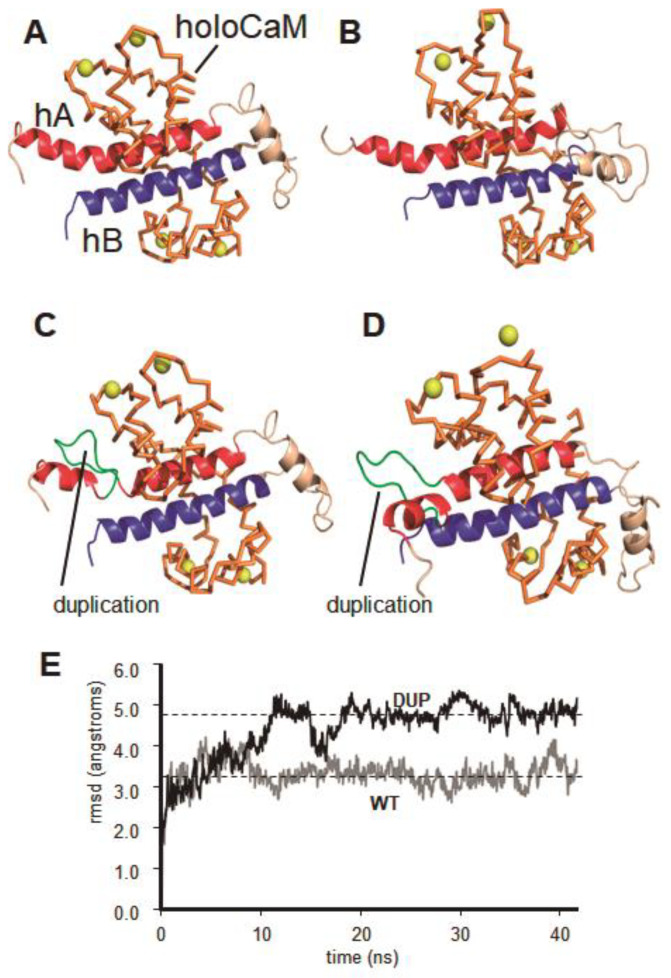
Analysis of unrestricted molecular dynamics simulation of the wild-type and variant calmodulin domain of the KCNQ2 proteins. (**A**) Structural model of the CaM binding domain of WT-KCNQ2 (helices hA in red, and hB in blue) in the presence of CaM in the calcium-saturated state (holoCaM, orange). Calcium ions are depicted as yellow spheres. (**B**) Same structure as in (**A**) after 40 ns of unrestricted molecular dynamics simulation. (**C**) Structural model of the CaM binding domain of the variant DUP-KCNQ2, containing the duplicated sequence (VQEQHRQKHFEK sequence, residues 320–331, green loop). (**D**) Same structure as in (**C**) after 40 ns of unrestricted molecular dynamics simulation. (**E**) Root mean square deviation (rmsd) values measured over the 40 ns molecular dynamics trajectories of the wild-type (WT-KCNQ2) and the variant (DUP-KCNQ2) structures. Note the higher variation of the rmsd values in the case of the DUP-KCNQ2 structure, caused by the presence of the duplicated sequence, while the WT-KCNQ2 structure does not change significantly.

This peptide insertion can affect the ion accessibility to the entrance of the channel by altering its properties, or alternatively, altering its regulation by calmodulin (CaM) and calcium. An increase in calcium resulted in its binding to CaM, which was responsible for the allosteric regulation of the transmembrane region of KCNQ2, which modulated the closing of the K^+^ channel [25,26,27]. The effect of the VQEQHRQKHFEK peptide duplication on the activity of the KCNQ2 channel was studied. For this, the structure modeling and molecular dynamics simulation was performed using 6FEH and 6FEG crystal three-dimensional structures [27]. The model of the duplicated peptide sequence suggests that the inserted amino acids form a loop in the helix hA of the CaM binding domain of KCNQ2, located in the vicinity of the C-lobe of CaM. Although the inserted sequence is located in the CaM binding domain, its presence does not represent a steric impediment to the interaction between the cytosolic domain of the channel and the CaM monomer. It has recently been described that a small change in the orientation of the helices hA and hB of KCNQ2 caused by a structural movement of CaM associated with the binding of two Ca^++^ ions in its C-lobe could be responsible for the allosteric regulation of the channel in response to an increase in the concentration of cytosolic calcium [20]. Dynamic modelling of the wild-type and variant KCNQ2 (DUP-KCNQ2) proteins was performed in response to increasing concentrations of calcium. The modelling was applied to the structural changes that the KCNQ2 protein underwent, mediated by CaM, when the calcium concentration was raised from basal cytosolic values (10–100 nM), in which Ca^++^ ions were bound only to the N-lobe of CaM, to high Ca^++^ values (1–10 μM) that also triggered the association of Ca^++^ to the CaM C-lobe. The position of hA and hB helices in WT-KCNQ2 modelled at high Ca2^++^ levels (Figure 3A) remained unchanged after 40 ns of free MD simulation (Figure 3B). In the case of the DUP-KCNQ2 model, at basal low Ca^++^ (Figure 3C), the organization was essentially identical to the WT- KCNQ2 model (Figure 3A). However, the presence of the duplicated sequence (green loop in Figure 3C) promoted a significant variation in the position of helix hA after 10 ns of free MD simulation (Figure 3E, black line) that was stably maintained after 40 ns of MD (Figure 3D, red). This new arrangement was equivalent to the structure of the same hA helix when Ca^++^ ions are bound only to the N-lobe of CaM [27]. In fact, the value of the root mean square deviation (rmsd), measured for helix hA under both conditions was 1.72 Å, indicating a virtually complete superposition. Since it has been proposed that the position of hA is responsible for the transmission of the high Ca^++^ signal from CaM to the K^+^ channel [27], this behavior predicts that this pathogenic KCNQ2 with the peptide insertion cannot respond to the increase in Ca^++^, and thus interferes with its regulation by CaM. This arrangement suggests that the variant protein, even in the presence of high concentrations of cytosolic calcium, can maintain a conformation equivalent to what it would have in the presence of basal calcium concentrations, and therefore would be functionally equivalent to a lack of response to calcium changes. Because this domain allosterically regulates the closure of the channel at high calcium concentrations, it suggests that within the variant protein, the channel could remain constitutively open, and would be unable to respond to the increase in calcium concentration in the cytoplasm and prevent its inhibitory effects, which would in turn lead to neuronal hyper-excitability.

## 3. Discussion

Known KCNQ2 pathogenic variants were very heterogeneous, and were distributed throughout the length of the protein (Figure 2). Pathogenic variants located within the transmembrane-pore domain altered the in- or out-flux of potassium ions, or their timing, resulting in a defective potassium transport. Other KCNQ2 pathogenic variants affected its interaction or regulation with calmodulin, or alternatively, facilitated the retention of the KCNQ2 protein in the endoplasmic reticulum, affecting its subcellular localization and leading to its reduction on the neuron surface [28]. The heterogeneity of KCNQ2 pathogenic variants suggests that what is important is the impairment of the normal KCNQ2 channel function via different mechanisms. KCNQ2 pathogenic variants alter, either quantitatively or qualitatively, the correct opening and closing of this potassium channel, resulting in a loss of function [27]. Despite their common loss of function, the associated neurological phenotype is clinically very heterogeneous. Therefore, whether the channel is locked in an open conformation or alters the balance between the in- and out-fluxes of potassium, the result is an alteration of its dampening signal on the sodium channel excitatory effects and a gain of neuronal excitability via the hyper-activation of sodium channels [28]. A reduction in the surface expression of KCNQ2 fails to suppress neuronal excitability [29]. Even KCNQ2 pathogenic variants having opposite functional effects on M potassium channels can produce similar EOEE neurological phenotypes [30]. Recently, a deletion in KCNQ2 was reported (c.913_915del [p.Phe305del)]) [31], which partially resembles the epileptic phenotype of this case, but without an associated cerebral palsy and distal neuromotor phenotype [31]. In murine heterozygous KCNQ2-knockout mice there was a lower seizure threshold [32]. KCNQ2-null mice have an increased excitability of primary sensory neurons, but do not have neuromotor alterations [33], and the inhibition of the Nav1.6 sodium channel counteracts the neuronal hyperexcitability [34]. Moreover, the replacement in murine embryos of KCNQ2 with the I250V mutant resulted in an increased neuronal excitability of pyramidal neurons [34].

There was a large KCNQ2 pathogenic variant heterogeneity in patients: 73 different mutated residues, including insertions and deletions, were detected in 172 mutant cases (Figure 2). However, there was no correlation between the locations of the pathogenic variants, despite their common loss of function, and the severity of the neurological phenotype. Moreover, there was no specific individual pathogenic variant associated to any specific phenotype, and the same pathogenic variant could be associated to different phenotypes, benign or severe, depending on the individual. The need to have additional genetic alterations to cause complex neurological phenotypes could explain why in cases in which the (c.628C>T; p.Arg210Cys) KCNQ2 pathogenic variant is inherited, the carrier parent has no, or mild, pathology and the affected offspring presents a very severe epileptic neurodevelopmental delay syndrome [35]. In a more recent study of novel mutations, there was no correlation between the location of mutation and the severity of phenotype [36]. This suggests that KCNQ2 alterations are necessary, but the severity and complexity of the neurological phenotype in each patient are a likely consequence of the contribution of additional gene variations in the patient genotype. The KCNQ2 pathogenic variant needs an environment in order to manifest [10]. A recent review on KCNQ2 [10] indicates the need for additional genetic events to explain the variability in the phenotype, which are currently unknown [10]. This means that both inherited and de novo KCNQ2 pathogenic variants require the cooperation of additional genetic alterations in order to be able to cause the full spectrum of the clinical phenotype [37].

We conclude that the genetic heterogeneity of early epileptic encephalopathy, cerebral palsy, and severe neurodevelopmental delays will have to be characterized in the context of the initiating pathogenic variant that is modulated by several additional genetic changes associated to neuronal functions, which remains to be identified. When unraveled, they might share common pathogenic pathways, although they might involve different genes. The identification of gene/protein networks associated to the clinical neurological phenotypes could set the basis for designing novel therapeutic approaches for the management of these patients, and minimize, or compensate, the neurological consequences of these pathogenic gene combinations.

## 4. Methods

### 4.1. Standard Protocol Approvals, Registrations, and Patient Consents

The genomic studies were performed for the diagnosis of a complex neurological syndrome of unknown origin. Total DNA was obtained from peripheral blood samples and used to perform the genomic diagnostic study. Both parents gave their informed consent for the genomic study.

### 4.2. Whole-Exome Sequencing

Whole-exome analysis (WES) was performed in the four members of the family with the affected boy as described in the Supplementary Methods. Briefly, 50 Mbp of captured exome content was selected for sequencing using Agilent SureSelect v5 and following the supplier’s Agilent SureSelect enrichment Protocol Guide. The generated DNA fragments (DNA libraries) were sequenced in the Illumina Hiseq 2000 platform with a sequence read length of 100 bp (Paired-End), obtaining an average coverage depth of 100 x in the target region. The Genome Reference Consortium Human Builds 37 and 38 (GRCh37 and GRCh38) were used as reference sequences for the identification of changes in the four family members. The exome analysis confirmed the paternity of the children. Sanger sequencing was used to confirm the mutations.

### 4.3. Analysis of Gene Alterations and Clinical Phenotype

All gene alterations or variants detected by WES were analyzed using the VarElect program [22] (LifeMap Sciences Inc., Tel Aviv, Israel) to search for correlations between the gene/protein biological function and the clinical phenotypes of the case, in order to detect a gene’s contribution to clinical symptoms.

### 4.4. Molecular Dynamics Simulation and Structural Modeling of KCNQ2

The 3D structures of the calmodulin (CaM) binding domain from the wild-type KCNQ2 channel (isoform 1, UniProtKB ID: O43526-1), and from the variant protein containing a duplicated VQEQHRQKHFEK sequence (residues 320–331) were modeled. As a template, the structure of the CaM binding domain of the human KCNQ2 channel was solved in the presence of CaM in the saturated calcium state (holoCaM/KCNQ2-hAB complex) or in the intermediate calcium state (intCaM/KCNQ2-hAB complex). The three-dimensional X-ray crystal structures of KCNQ2, 6FEH, and 6FEG are available in the Protein Data Bank [27].

Models of normal KCNQ2 and its peptide-duplication mutant were built using the SWISS-MODEL server (http://swissmodel.expasy.org), and their structural quality was within the range of those accepted for homology-based structures (Anolea/Gromos/QMEAN4). Modeled structures were subjected to MD simulation using the AMBER14 molecular dynamics package (University of California, San Francisco), as previously reported [38].

The 3D structures were solved with periodic octahedral pre-equilibrated solvent boxes using the LEaP module of AMBER, with 12 Å as the shortest distance between any atom in the protein and the periodic box boundaries. Free MD simulations were performed essentially as previously described [38], using the PMEMD program of AMBER and the ff14SB force field (University of California San Francisco). The SHAKE algorithm was used, allowing for a time step of 2 fs. Systems were initially relaxed over 15,000 steps of energy minimization with a cut-off of 12 Å. Simulations were then started with a 20 ps heating phase, raising the temperature from 0 to 300 K in 10 temperature-change steps, after each of which velocities were reassigned. During minimization and heating, the Cα trace dihedrals were restrained with a force constant of 500 kcal mol^−1^ rad^−2^ and gradually released in an equilibration phase in which the force constant was progressively reduced to 0 over 200 ps. After the equilibration phase, 40 ns of unrestricted MD simulations were obtained for wild-type and variant structures. Root mean square deviation (rmsd) of Cα trace was monitored along the trajectories, and was measured every 20 ps. MD trajectories were analyzed using VMD software [39]. Figures were generated using the Pymol Molecular Graphics System (Schrödinger, LLC).

## Data Availability

The *KCNQ2* partial duplication has been deposited in the ClinVar database. (https://www.ncbi.nlm.nih.gov/clinvar/variation/617505). Whole Exome Sequence (WES) raw data files are available in: https://www.ncbi.nlm.nih.gov/sra/PRJNA629061 https://www.ncbi.nlm.nih.gov/bioproject/PRJNA629061 https://digital.csic.es/handle/10261/170405.

## Supplementary Materials

Supplementary materials can be found at https://www.mdpi.com/1422-0067/21/12/4447/s1.
