# Supplementary material for: Novel Dominant KCNQ2 Exon 7 Partial In-Frame Duplication in a Complex Epileptic and Neurodevelopmental Delay Syndrome
Source: Int J Mol Sci. 2020 Jun 23;21(12):4447. doi: 10.3390/ijms21124447 (PMC7352878; doi:10.3390/ijms21124447)
Supplement: Supplementary file 1 [file ijms-21-04447-s001.pdf]

Supplementary Methods

EXOME SEQUENCING Information

Type of Read: Paired-end
Read Length: 101
Number of Samples: 4
Library Kit: SureSelectXT Library Prep Kit
Library Protocol: SureSelectXT Target Enrichment System for Illumina Version B.2, April2015
Type of Sequencer HiSeq 2000

DNA quality control:
Electrophoresis in gel agarose (1,5%) in TAE Buffer.
Fluorescence based DNA quantification in Qubit Fluorometer.

Generation of raw WES data:
The Illumina Hiseq generated raw images utilizing HCS (HiSeq Control Software v2.2) for system control and base calling through an integrated primary analysis software called RTA (Real Time Analysis. v1.18). The BCL (base calls) binary was converted into FASTQ utilizing illumina package bcl2fastq (v1.8.4).

Raw data statistics:
The total number of bases, reads, GC (%), Q20 (%), and Q30 (%) were calculated for the 4 samples.
For example, in CDC-PAD, 104,544,694 reads were produced, and total read bases were 10.6G bp. The GC content (%) was 47.87% and Q30 was 91.87%.

Table 1. Raw data Stats

Sample ID	Total read bases (bp)	Total reads	GC(%)	AT(%)	Q20(%	Q30(%
CDC-PAD	10,559,014,094	104,544,694	47.87	52.13	95.71	91.87
CDC-MAD	10,898,803,344	107,908,944	47.79	52.21	95.73	91.88
CDC-HO	10,564,626,462	104,600,262	47.91	52.09	95.95	92.22
CDC-HA	10,065,966,434	99,663,034	47.75	52.25	95.93	92.22

- Sample ID: Sample name.
- Total read bases: Total number of bases sequenced.
- Total reads: Total number of reads. In illumina paired-end sequencing, read1 and read2 are added.
- GC(%): GC content.
- AT(%): AT content.
- Q20(%: Ratio of reads that have phred quality score of over 20.
- Q30(%) Ratio of reads that have phred quality score of over 30.

Exome data analysis:
Human genome reference: GRCh38
Alignment, variant calling and filtering with CLC Genomics Workbench v7.5.2

Supplementary Table S1

DE NOVO MUTATIONS IN AFFECTED CHILD (Heterozygous)				
**GENE**	**Aminoacid change**	**Mutation Position**	**Frequency Variant/alleles (ExAC) [1]**	**Inheritance**
KCNQ2	Partial exon 7 duplication	This report		De novo heterozygosis
MPPED1		22_43831052_C/T		De novo
**INHERITED DE NOVO MUTATIONS IN AFFECTED CHILD (Heterozygous)**				
RARS2	S443P	Exon 16, c.1327T>C	5/121272	Father heterozygosis

RECESSIVE gene variants IN AFFECTED CHILD (Homozygous)				
**Gene**	**Aminoacid change**	**Presentation**	**Variant ID**	**Potential effect**
ZNF398	R8Q	Intronic variant	rs917123	Damaging "low confidence"
KLHL38	C504Y	homozygous	rs11779866	damaging
NPBWR1	Y135F	homozygous	rs33977775	damaging
SCARF1	I510I or S498L	Variant in aberrant message	rs34849297	damaging
LIPF	T161A	homozygous	rs814628	damaging
OR51B6	N40S<br>I90T<br>T131I<br>L172F<br>S275R		rs4910756<br>rs7483122<br>rs5006886<br>rs5006884<br>rs5024042	damaging
AKIP1	T170M, T143M	Intron variant	rs2016844	damaging
ANXA11	R230C		rs1049550	damaging
NPNT	Q189H		rs35132891	damaging
C7orf31	A158T		rs12535348	damaging
OR1D2	T240I	homozygous	rs4300683	damaging
OR51B5	I102T		rs11036912	damaging
CRYBG3	N926H		rs4857302	damaging
SGSM2	R374Q		rs2248821	damaging
SLC22A18	R12Q	homozygous	rs1048047	Damaging
OR5H14	G64R		rs4241468	damaging
TMEM71	Upstream variant		rs1895807	Damaging, low confidence
NAALADL2	P622R	homozygous	rs9866564	Damaging

[1] K.J. Karczewski, B. Weisburd, B. Thomas, M. Solomonson, D.M. Ruderfer, D. Kavanagh, T. Hamamsy, M. Lek, K.E. Samocha, B.B. Cummings, D. Birnbaum, C. The Exome Aggregation, M.J. Daly, and D.G. MacArthur, The ExAC browser: displaying reference data information from over 60 000 exomes. Nucleic Acids Res 45 (2017) D840-D845.

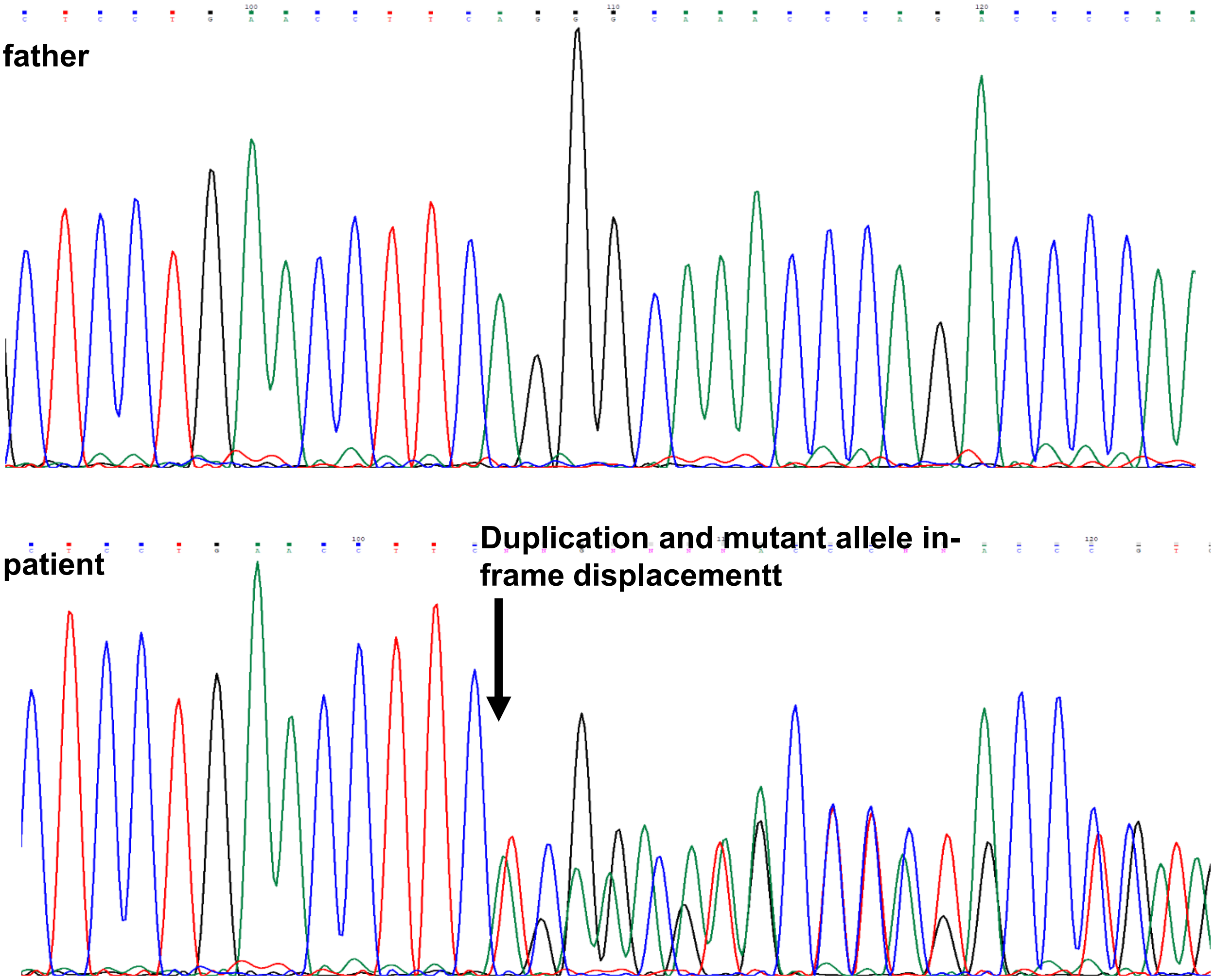

Supplementary Figure S1. Sanger sequence showing the normal allele (top chromatogram) and the allele in the patient (lower chromatogram) with the location (arrow) of the duplication one allele. From this position there is an overlap of the two alleles sequences.

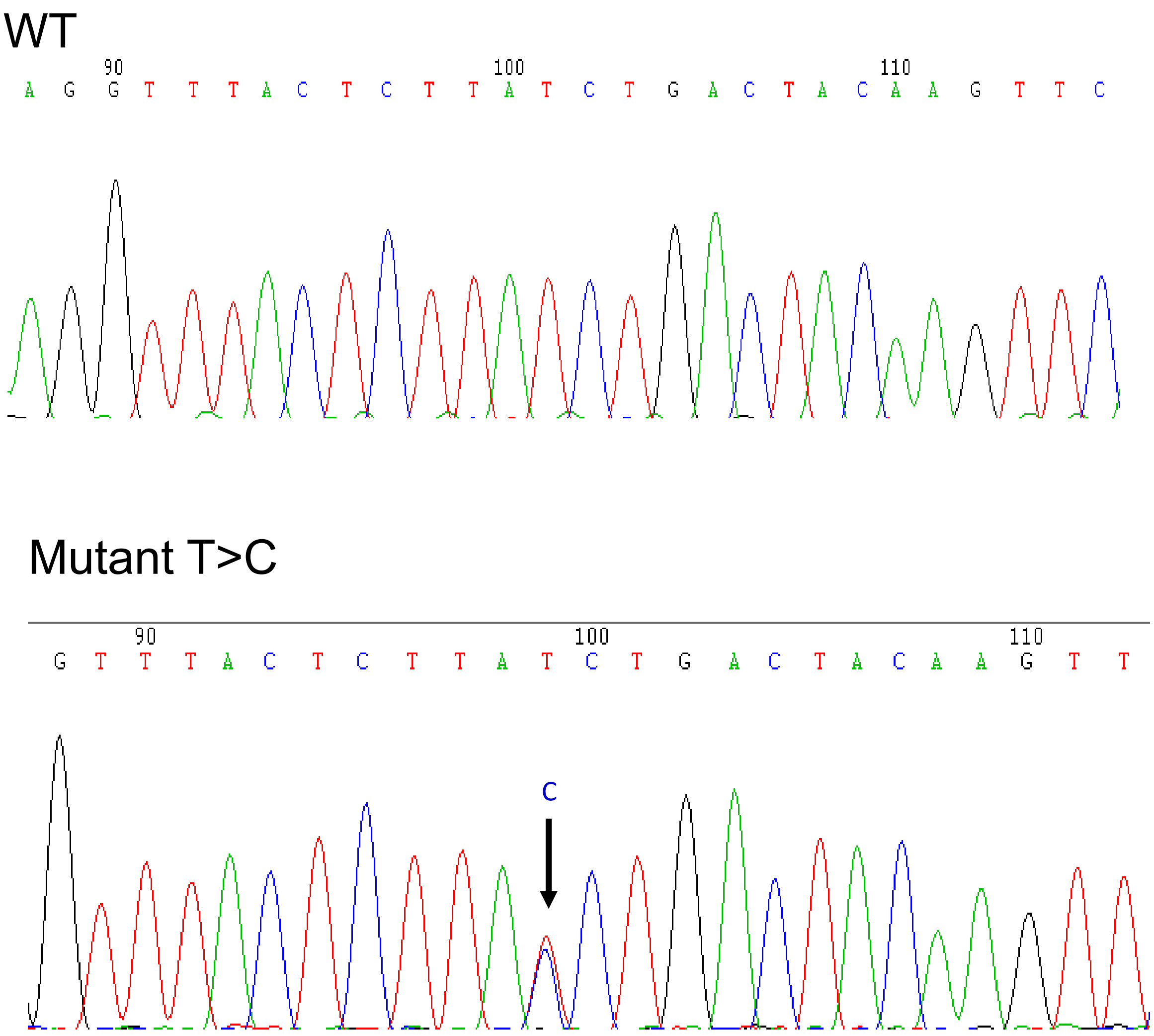

Supplementary Figure S2. Sanger sequence of the *RARS2* gene showing the normal allele (top chromatogram) and the allele in the patient (lower chromatogram) with the location (arrow) of the nucleotide substitution (T > C).
